# *CHKA *and *PCYT1A *gene polymorphisms, choline intake and spina bifida risk in a California population

**DOI:** 10.1186/1741-7015-4-36

**Published:** 2006-12-21

**Authors:** James O Ebot Enaw, Huiping Zhu, Wei Yang, Wei Lu, Gary M Shaw, Edward J Lammer, Richard H Finnell

**Affiliations:** 1 Center for Environmental and Genetic Medicine, Institute of Biosciences and Technology, Texas A&M University System Health Science Center, Houston, Texas 77030, USA; 2 California Birth Defects Monitoring Program, Berkeley, CA, USA; 3 Children's Hospital Oakland Research Institute, Oakland, CA, USA; 4 Center for Environmental and Rural Health, Texas A&M University, College Station, Texas 77843, USA

## Abstract

**Background:**

Neural tube defects (NTDs) are among the most common of all human congenital defects. Over the last two decades, accumulating evidence has made it clear that periconceptional intake of folic acid can significantly reduce the risk of NTD affected pregnancies. This beneficial effect may be related to the ability of folates to donate methyl groups for critical physiological reactions. Choline is an essential nutrient and it is also a methyl donor critical for the maintenance of cell membrane integrity and methyl metabolism. Perturbations in choline metabolism *in vitro *have been shown to induce NTDs in mouse embryos.

**Methods:**

This study investigated whether single nucleotide polymorphisms (SNPs) in human choline kinase A (*CHKA*) gene and CTP:phosphocholine cytidylytransferase (*PCYT1A*) gene were risk factors for spina bifida. Fluorescence-based allelic discrimination analysis was performed for the two *CHKA *intronic SNPs hCV1562388 (rs7928739) and hCV1562393, and *PCYT1A *SNP rs939883 and rs3772109. The study population consisted of 103 infants with spina bifida and 338 non-malformed control infants who were born in selected California counties in the period 1989–1991.

**Results:**

The *CHKA *SNP hCV1562388 genotypes with at least one C allele were associated with a reduced risk of spina bifida (odds ratio = 0.60, 95%CI = 0.38–0.94). The *PCYT1A *SNP rs939883 genotype AA was associated with a twofold increased risk of spina bifida (odds ratio = 1.89, 95% CI = 0.97–3.67). These gene-only effects were not substantially modified by analytic consideration to maternal periconceptional choline intake.

**Conclusion:**

Our analyses showed genotype effects of *CHKA *and *PCYT1A *genes on spina bifida risk, but did not show evidence of gene-nutrient interactions. The underlying mechanisms are yet to be resolved.

## Background

Neural tube defects (NTDs) are complex congenital malformations of the central nervous system. Anencephaly and spina bifida are the most common and severe forms of NTDs. The birth prevalence of NTDs varies from approximately 0.8/1,000 births in many areas of the US to 3.5/1,000 in Mexico [1,2]. Epidemiologic studies suggest that both genetic and environmental factors contribute to NTD etiologies. Although most factors appear to explain very little of the population burden of NTDs, maternal nutritional factors do appear to substantially contribute to the complex etiologies of NTDs. Foremost among these factors has been the role of periconceptional intake of folic acid in reducing recurrence and occurrence risks of women for NTD-affected pregnancies [3-11].

Nutrients and nutrition-related factors other than folic acid have been observed to influence NTD risks. For example, increased intakes of methionine, zinc, vitamin C, and choline have been associated with reduced NTD risk [12-15]. With respect to choline, it was recently observed that increased periconceptional intakes of diets with choline were associated with reduced risks of NTD-affected pregnancies that were independent of maternal folate intakes.[15] This observation provided evidence to suggest that deficiencies in methyl donors may be associated with NTD risk, that is to say, a less than optimal methyl-donor supply and DNA methylation status has been a suggested area for research efforts for certain birth defects [16]. Choline, like folate, is a methyl donor in the methylation of homocysteine to methionine [17,18].

Choline is utilized for the *de novo *synthesis of phosphatidylcholine (PC) and sphingomyelin through the cytidine diphosphocholine (CDP-choline) pathway. There are three reactions in this pathway. The first reaction is catalyzed by the enzyme choline kinase (CHK; ATP:choline phosphotransferase, EC 2.7.1.32), which phosphorylates choline by donating an ATP [19]. The second reaction involves phosphocholine (P-Cho) cytidylyl transferase (CCT), which catalyzes the formation of CDP-Choline from P-Cho and CTP [20]. The final reaction uses choline phosphotransferase (CPT), which catalyzes the condensation reaction of CDP-Choline with diacylglycerol [21]. Phosphatidylcholine (PC) and sphingomyelin are required for maintaining cell membranes and play important roles in regulation of cell growth, differentiation, and death through the production of diacylglycerol (DAG) and ceramide (CER), which are cell signaling molecules [22,23].

In gastrulation- and neurulation-stage mouse embryos, choline was elucidated to be used primarily for PC synthesis favoring the CDP-choline pathway, although some betaine and acetylcholine was also generated [24]. Using the choline uptake inhibitor 2-dimethlyaminoethanol (DMAE) and an inhibitor of PC synthesis, 1-O-octadecyl-2-O-methly-rac-glycerol-3-phosphocholine (ET-18-OCH_3_), Fisher and co-workers observed an increase in cell death and both craniofacial and NTDs in neurulation stage mouse embryos grown in culture [24].

In humans, choline kinase has two isoforms, CHKα and CHKβ, with the α as a dominant isoform. The *CHKA *gene encoding choline kinase α is located at chromosome 11q13.2. Our study focused on the *CHKA *gene. *PCYT1A *and *PCYT1B *encode CCTα and CCTβ, respectively. *PCYT1A *located at chromosome 3q29, while *PCYT1B *is located at chromosome Xp22.11 [25,26]. In this study we focused on *PCYT1A *gene.

Given that periconceptional intake of choline has been associated with decreased risk of NTD-affected pregnancies [15], we investigated *CHKA *and *PCYT1A *genotypes on risk of spina bifida. We also investigated these genotypes in combination with lowered maternal intake of choline as risk factors of spina bifida.

## Methods

### Study population

Data investigated were derived from a case-control study that previously described a risk reduction in NTDs associated with maternal periconceptional intake of choline. In brief, these data were derived from the California Birth Defects Monitoring Program, a population-based active surveillance system for collecting information on infants and fetuses with congenital malformations [27]. Births occurring in selected California counties in the period 1989–1991 were eligible for the original case-controlled interview study. For the current investigation, we identified 103 spina bifida infants whose newborn screening blood specimen could be obtained and whose mothers' choline intake was estimated. As controls, we identified 338 non-malformed control infants whose newborn screening blood specimen could be obtained and whose mothers' choline intake was estimated. Among the 103 cases, 36% were non-Hispanic whites, 51% were Hispanics, and 13% were of other race/ethnic background. Among the 338 controls, 56% were non-Hispanic whites, 25% were Hispanics, and 19% were of another race/ethnic background. All samples were obtained with the approval from the state of California Health and Welfare Agency Committee for the Protection of Human Subjects. Genomic DNA used for genotyping was collected from newborn screening blood spots and extracted according to the Puregene Genomic DNA Extraction kit (Gentra, Minneapolis, MN, USA) protocol.

### Genotyping procedure

Two intronic *CHKA *SNPs, hCV1562388 (A>C) and hCV1562393 (C>G) as well as two intronic *PCYT1A *SNPs, rs939883 (T>A) and rs3772109 (T>C) were selected as tagging SNPs using SNPBrowser software (v2.0) (Applied Biosystems, Foster City, CA, USA). hCV1562388 and hCV1562393 cover a 10 kbp genomic region of the *CHKA *gene; rs939883 and rs3772109 cover a 40 kbp genomic region of the *PCYT1A *gene. Samples were genotyped using a fluorescence-based allelic discrimination assay on an ABI PRISM^® ^7900HT sequence detection system (Applied Biosystems, Forster City, CA, USA), following the manufacturer's protocol. These intronic SNPs were selected based on the assumption that they might be in linkage disequilibrium (LD) with disease-causing variation. Primers and fluorescent dye labeled probes were purchased from ABI as Assay-on-Demand reagents. The Assays-on-Demand SNP genotyping consisted of a 20 × mix of unlabeled PCR primers and TaqMan^**® **^probe labeled with FAM™ and VIC™ fluorescent dyes. The FAM™ dye is linked to the 5' end of one allele in the probe while the VIC™ dye is linked to the 5' end of the other allele in the probe. These dyes are used for allelic discrimination of each SNP.

Allelic discrimination PCR reactions were performed on 384-well plates. Each reaction contained 2.5μL TaqMan Universal PCR Master Mix, No Amp Erase^® ^UNG (2 ×), 0.25 μL of 20 × Assay-on-Demand™ SNP Genotyping Assay Mix, 2.25μL gDNA (1–20 ng) diluted in dH_2_O making up a total volume of 5 μL per reaction. The thermocycling conditions started with a denaturation step at 95°C for 10 min, followed by 45 cycles of denaturation at 92°C for 15 sec, annealing and extension at 60°C for 1 minute. Results were read and interpreted blind as to case/control status, and each assay was performed in duplicate.

### Statistical analysis

Deviation from Hardy-Weinberg Equilibrium among control infants was evaluated by a chi-square test. Odds ratios (ORs) and associated 95% confidence intervals (95% CIs) were used to measure associations between infant *CHKA, PCTY1A *genotypes or compound genotypes, and spina bifida risk. For genotype comparisons, homozygous wild-type infants served as the reference group to which heterozygotes and variant homozygotes were compared. Choline intake values were considered according to quartile cutoffs. For quartile analyses, odds ratios and 95% CI were computed to estimate risk using the lowest quartile as the reference. All statistical analyses for this study were performed using SAS software v9.1 (SAS Institute Inc, Cary, NC, USA). Samples failed the genotyping assay were excluded for statistic analyses.

## Results

Table 1 shows the previously observed association in this dataset between choline intake and, specifically, spina bifida risk. That is, ORs indicated that maternal intakes of choline in the periconceptional period were associated with reduced risk.

Genotyping results of all SNPs were in Hardy-Weinberg Equilibrium (HWE) among controls (χ^2 ^test: P > 0.05). Among non-Hispanic white and Hispanic white, the minor allele frequencies (MAF) were 0.39 and 0.27 for hCV1562388, 0.20 and 0.12 for hCV1562393, 0.32 and 0.33 for rs939883, 0.38 and 0.47 for rs3772109, respectively. Linkage disequilibrium (LD) were evaluated by D' and r^2 ^using the Haploview program. For *CHKA *gene, hCV1562388 and hCV1562393 are in complete LD in study population (D' = 0.91, r^2 ^= 0.065). For *PCYT1A *gene, D' for rs939883 and rs3772109 was 0.81 and r^2 ^was 0.29.

Table 2 shows 'gene-only' effects associated with *CHKA *SNPs. These data showed a reduced risk of spina bifida for individuals with either one or more C alleles for the SNP hCV1562388 (A>C) but not with SNP hCV1562393(C>G). Infants with AA genotype for *PCYT1A *SNP rs939883 showed a nearly twofold increased risk of spina bifida relative to those with the TT genotype, however, it is not statistically significant and may be caused by chance.

Table 3 shows results of analyses that investigated gene-nutrient effects, that is to say, combined effects on risk of spina bifida between maternal choline intake and homozygous genotypes. We did not observe evidence of a gene-nutrient interaction between *CHKA *SNPs and maternal periconceptional choline intake. The increased risk for *PCYT1A *observed in gene-only analyses did not appear to be further influenced by maternal choline intake.

## Discussion

This study investigated an underlying genetic explanation for a previously identified association between choline intake and spina bifida risk [15]. In the current study, we investigated intronic gene variants of two enzymes involved in the metabolism of dietary choline via the CDP-choline pathway. We believe this is the first study to evaluate DNA sequence variants in the human *CHKA *and *PCYT1A *genes for a possible association with NTD risk. Reduced risks of spina bifida were found for *CHKA *SNP hCV1562388, and increased risks were found for SNP rs939883. These risks, however, were not modified by maternal periconceptional intake levels of dietary choline. Thus, our study showed gene-only effects but did not observe gene-nutrient interaction effects associated with choline intake. The results indicate that dietary choline and choline metabolism genes may affect spina bifida risk independently or through some other unknown mechanisms. This interpretation should be taken cautiously owing to limited statistical power; if gene-only effects are true, a lack of gene-nutrient interaction effects may be due to small sample sizes and limited statistical power.

The functional impacts of the *CHKA *SNP hCV1562388 (A>C), *CHKA *SNP hCV1562393(C>G), *PCYT1A *SNP rs939883 and SNP rs3772109, or other sequence variations associated with these tagging SNPs are unknown with respect to choline, PC, or homocysteine concentrations. Unlike the enzymes cystathionine beta-synthase (*CBS*), methionine synthase reductase (*MTR*), and 5,10-methylenetetrahydrofolate reductase (*MTHFR*), that are directly involved in folate-homocysteine metabolism, the CHK metabolic profile may be modulated, as its effect is transmitted through the choline pathway [24].

This study had limitations that lessen our ability to make solid inferences from the results. As noted, the study had limited sample size; therefore, precision was low for many of the estimated effects, particularly those involving gene-nutrient interactions. Our study, explored only four tagging SNPs in two genes, limiting the ability to detect possible genetic modifiers related to choline metabolism.

## Conclusion

Despite its limitations, this study provided initial data indicating a potential association between *CHKA *and *PCYT1A *gene variants and spina bifida risk. Future studies of additional SNPs within the *CHKA *and *PCYT1A *genes should be investigated as potential predictors of spina bifida risks.

## Competing interests

The author(s) declare that they have no competing interests.

## Authors' contributions

JOE performed the majority of the experiments and wrote the manuscript; HPZ designed, optimized and performed part of the experiments and helped with manuscript writing; WY performed the statistic analyses; WL performed part of the experiments and helped with manuscript writing; GMS performed epidemiology study design and helped with manuscript writing; EJL helped with study design and manuscript writing; RHF performed experimental design and helped with manuscript writing.

## Pre-publication history

The pre-publication history for this paper can be accessed here:



## Figures and Tables

**Table 1:** Effect estimates (odds ratio) for spina bifida-affected pregnancies associated with maternal choline intake during the periconceptional period, California 1989–1991. OR: odds ratio; CI: confidence interval.

Total choline intake (mg/day)	Cases	Controls	OR	95% CI
	103	338		
Quartile Measure				
≤ 289.93	34	85	Reference	
289.93–372.01	22	84	0.66	0.35–1.21
372.02–513.24	27	85	0.79	0.44–1.43
> 513.24	20	84	0.60	0.32–1.12

**Table 2:** Effect estimates (odds ratios) for spina bifida-affected pregnancies associated with *CHKA *SNPs hCV1562388 (A>C) and hCV1562393(C>G), *PCYT1A *SNPs rs939883 (T>A) and rs3772109 (T>C), California 1989–1991. OR: odds ratio; aOR: adjusted odds ratio by maternal ethnicity; CI: confidence interval.

*CHKA *gene	Cases (%)	Controls (%)	OR (95% CI)	aOR (95% CI)
SNP hCV1562388 (A>C)				
AA	54 (54.0)	138 (41.2)	Reference	-
AC	38 (38.0)	154 (46.0)	0.63 (0.39–1.01)	0.62 (0.39–1.00)
CC	8 (8.0)	43 (12.8)	0.48 (0.21–1.08)	0.48 (0.21–1.10)
AC+CC	46 (46.0)	197 (58.8)	0.60 (0.38–0.94)	0.55(0.38–0.99)
SNP hCV1562393(C>G)				
CC	66 (67.3)	225 (67.6)	Reference	-
CG+GG	32 (32.6)	108 (32.4)	0.99 (0.61–1.62)	1.01 (0.62–1.66)

*PCYT1A *gene	Cases (%)	Controls (%)	OR (95% CI)	aOR (95% CI)

SNP rs939883 (T>A)				
TT	35 (34.3)	132 (39.9)	Reference	-
TA	48 (47.1)	161 (48.6)	1.12 (0.69–1.84)	1.14 (0.70–1.87)
AA	19 (18.6)	38 (11.5)	1.89(0.97–3.67)	1.85 (0.95–3.61)
SNP rs3772109 (T>C)				
TT	33 (33.7)	104 (31.2)	Reference	-
TC	51 (52.0)	164 (49.2)	0.98 (0.59–1.62)	0.99 (0.60–1.65)
CC	14 (14.3)	65 (19.5)	0.68 (0.34–1.36)	0.69 (0.34–1.41)

**Table 3:** Effect estimates (odds ratios) for spina bifida-affected pregnancies associated with maternal choline intake, *CHKA *SNP hCV1562388 (A>C), hCV1562393(C>G), *PCYT1A *SNPs rs939883 (T>A) and rs3772109 (T>C) genotypes. Breslow-Day test: P > 0.05. Lower 25%: total choline intake ≤ 289.93 mg/day. Higher 75 %: total choline intake > 513.24 mg/day.

*CHKA *gene	Cases	Controls	OR	(95% CI)
SNP hCV1562388 (A>C)				
Lower 25 percentile			Reference	
AA	17	35		
AC + CC	15	49	0.63	0.28–1.63
Higher 75 percentile			Reference	
AA	37	103		
AC + CC	31	148	0.58	0.34–1.00
SNP hCV1562393(C>G)				
Lower 25 percentile			Reference	
CC	22	58		-
CG + GG	9	25	1.08	0.43–2.70
Higher 75 percentile			Reference	
CC	44	167		-
CG + GG	23	83	1.05	0.60–1.86

*PCYT1A *gene	Cases	Controls	OR	95% CI

SNP rs939883 (T>A)				
Lower 25 percentile			Reference	
TT	9	37		
TA	17	35	1.23	0.43–3.52
AA	7	11	1.66	0.41–3.52
Higher 75 percentile			Reference	
TT	26	95		
TA	31	126	0.90	0.50–1.61
AA	12	27	1.62	0.72–3.64
SNP rs3772109 (T>C)				
Lower 25 percentile			Reference	
TT	9	27		
TC	17	40	1.28	0.50–3.28
CC	5	16	0.94	0.27–3.29
Higher 75 percentile			Reference	
TT	24	77		
TC	34	124	0.88	0.49–1.59
CC	10	49	0.59	0.25–1.37
